# Landscape analysis of pre-registered clinical trials involving classical psychedelics

**DOI:** 10.1177/02698811251371690

**Published:** 2025-10-21

**Authors:** Abdo Uyar, Linda Forbrich, Ulrike Lueken, Ricarda Evens

**Affiliations:** 1 Department of Psychology, Humboldt-Universität zu Berlin, Germany; 2 German Center for Mental Health (DZPG), Partner Site Berlin-Potsdam, Germany

**Keywords:** psychedelics, clinical trials, pre-registration protocols, psychedelic research, landscape analysis, review, trends

## Abstract

Psychedelic clinical research is expanding rapidly. This review analyses the state and trends in psychedelic clinical trial registrations. A systematic search of ClinicalTrials.Gov was conducted on 11 November 2024, to identify registered interventional trials investigating (therapeutic) effects of serotonergic psychedelics (e.g. lysergic acid diethylamide [LSD], psilocybin, [5-MeO-]DMT). Analyses included a negative binomial regression to assess time trends and descriptive summaries of study characteristics. Outcomes included registration trends, substance distribution, study phase progression, sample and trial characteristics, geographical distribution and psychotherapy reporting. A total of 241 trials were identified, with registrations rising exponentially after 2006 and an acceleration post-2019. Two-thirds of trials are ongoing or planned. Psilocybin remains the most frequently studied substance and is most advanced towards approval, but short-acting psychedelics ([5-MeO-]DMT) have recently been introduced with a more focused clinical scope. Industry involvement is increasing, though university-led research still dominates. Reports of psychotherapy components increased following 2023 FDA recommendations, though no major improvements in intervention descriptions were observed. The rapid expansion of registered psychedelic clinical trials with diverse indications and substances reflects growing clinical interest. While university-led studies initiated early investigations and established a broad knowledge base, later industry involvement increasingly prioritizes scalability and economic considerations by adopting a focused approach towards clinical approval. Inconsistent reporting of psychotherapeutic components limits cross-study comparability and complicates systematic investigations into which combinations of therapeutic elements (type, timing, intensity) may optimize clinical outcomes. Future efforts should focus on complete and standardized trial reporting at study registration to minimize bias, reduce interpretative ambiguity and facilitate cross-trial comparisons.

## Introduction

After decades of dormancy, clinical research on serotonergic psychedelics resurged in the 1990s with investigator-initiated dose–response studies on N,N-dimethyltryptamine (DMT), a short acting psychdelicsubstance, in healthy volunteers (Strassman et al., 1994), followed by trials exploring the therapeutic potential of psilocybin and lysergic acid diethylamide (LSD), which are longer acting and widely used psychedelic sustances (Grob et al., 2011). Pioneering research at Johns Hopkins University, Imperial College London and New York University has provided promising evidence for mental health benefits of psychedelics among healthy participants (Carhart-Harris et al., 2012; Griffiths et al., 2006), and later in conditions such as nicotine dependence (Johnson et al., 2014), anxiety and depression in patients with life-threatening illnesses (Ross et al., 2016), or depression (Carhart-Harris et al., 2016). In recent years, the field of clinical research involving psychedelics has expanded (e.g. Yao et al., 2024), with increasing numbers of larger-scale randomized controlled trials (Goodwin et al., 2022; Raison et al., 2023; Von Rotz et al., 2023).

Early results stimulated a rise in biotech companies researching and developing psychedelic therapies. These companies have largely been supported by venture capital, and nearly 50 have since become publicly traded (Lenz, 2022). Psychedelic start-up investments peaked at $2 billion in 2021 but dropped to $526 million in 2022 due to economic uncertainty (Aday et al., 2023). Yet, the psychedelic market is speculated to reach $10.75 billion by 2027, potentially outpacing the U.S. cannabis market (Phelps et al., 2022). Initially hesitant, large pharmaceutical companies show growing interest through recent partnerships (e.g. Otsuka Pharmaceutical’s collaboration with Mindset Pharma).

The fields’ increasing complexity presents challenges in tracking advancements and maintaining a comprehensive overview. ClinicalTrials.Gov serves as a crucial resource for monitoring research progress in this evolving landscape. Launched in 2000, it is the largest registry of publicly and privately funded clinical trials worldwide, detailing objectives, sample characteristics, locations and outcomes (McCray and Ide, 2000; Ogino et al., 2014). Unlike delayed journal publications, the analysis of pre-registration protocols provides up-to-date data on planned, ongoing and completed trials, helping researchers track the current landscape and anticipate findings.

Clinical trial registration combats publication bias and selective reporting (Dickersin and Rennie, 2012), is legally required under the FDA Amendments Act and must be regularly updated by study sponsors (U.S. National Library of Medicine, 2024). Since 2005, the International Committee of Medical Journal Editors has mandated pre-registration to be considered for publication (Dickersin and Rennie, 2012). Likewise, the Declaration of Helsinki requires pre-registration before the start of participant recruitment (World Medical Association, 2008).

Previous research has explored the psychedelic landscape through pre-registration protocols with varying scopes (Kurtz et al., 2022; Norring and Spigarelli, 2024; Siegel et al., 2021); Norring and Spigarelli (2024), for example, discussed how inefficiencies in trial designs and the simultaneous investigation of different indications in studies with psilocybin may hinder progress towards market approval. Given the fields’ rapid expansion, previous findings, however, may no longer reflect the current state of the field or are restricted to specific psychedelic substances. Furthermore, previous analyses show limitations in search strategies such as the lack of inclusion of proprietary or brand names of substances, omission of Boolean operators (e.g. ‘*’) or restrictions to specific mental health conditions.

The present analysis addresses previous limitations by systematically capturing clinical trials on all classical serotonergic psychedelics (i.e. psilocybin, LSD, DMT, 5-MeO-DMT and mescaline), by employing a comprehensive search strategy, unrestricted by specific mental disorders, and including substance (brand) name variants (e.g. COMP360). By analysing pre-registration data, this analysis provides an up-to-date systematic overview of the research landscape. It offers insights into the substances most commonly studied, conditions targeted, and geographic and institutional distributions of research efforts. It identifies current trends and methodological approaches, with a specific emphasis on the integration of psychotherapy within psychedelic trials.

## Methods

### Search strategy

To capture the current landscape and give a prospect about future trends, a search was conducted on 11 November 2024, using ClinicalTrials.Gov’s expert search function. The search string, detailed in Supplemental Material A, includes generic terms, specific chemical and patented names. Brand names were retrieved through Psychedelic Alpha’s Drug Development Tracker (Haichin, n.d.) and pharmaceutical company websites.

### Study selection and eligibility criteria

**Figure 1.**
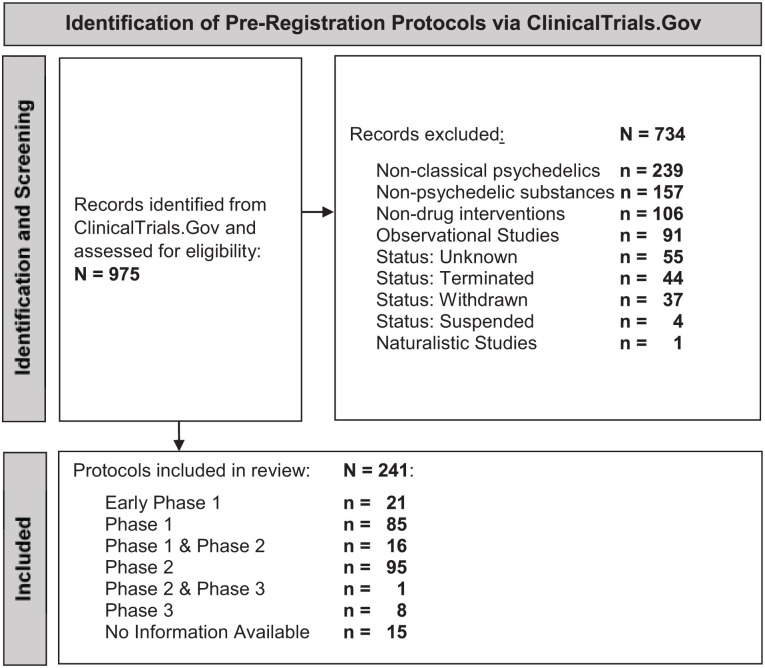
Flow chart of pre-registration protocols identified and included.

Completed, active and upcoming interventional clinical trials involving classical, serotonergic psychedelics of all dosages (including microdosing studies) were included. Observational or naturalistic studies were excluded. Classical psychedelics are commonly defined as 5-HT2A receptor agonists that induce altered states of consciousness. Atypical substances such as cannabis, 3,4-Methylenedioxymethamphetamine (MDMA) or ketamine were excluded due to their distinct pharmacological profiles and divergent subjective effects. Compounds sometimes referred to as ‘non-hallucinogenic psychedelics’ or ‘neuroplastogens’ were not included, as their acute subjective effects do not align with the classical psychedelic experience. Protocols with Suspended (paused with possible resumption), Terminated (stopped with no intent to resume), Withdrawn (ceased before enrolling participants) or Unknown status (unverified for over 2 years after specified completion date; U.S. National Library of Medicine, n.d.) were excluded (Figure 1).

### Data extraction and data pre-processing

Variables were directly downloaded whenever possible or extracted utilizing a two-extractor approach (Supplemental Material B). Discrepancies in extraction were resolved by re-review and discussion. Sponsor type data downloaded from ClinicalTrials.Gov were limited to a binary classification of ‘industry’ and ‘others’ (encompassing all non-industry entities). Thus, sponsor types were manually re-coded into four categories: university, industry (for-profit; e.g. *atai Therapeutics, Inc*., Amstelveen, The Netherlands), organization (non-profit; e.g. *Centre for Addiction and Mental Health*), individual person or combinations in collaborative sponsorships. Clinical indications were manually and inductively categorized into 10 broader categories: Affective Disorder, Substance-Related Disorder, Psychiatric Symptoms in Life-Threatening Disease, Pain-Related Disorder, Trauma- and Stressor-Related Disorder, Obsessive-Compulsive Disorder, Eating Disorder, Anxiety Disorder, Mixed Disorders, Other (Figure 6). Protocols with multiple sub-studies were classified under each relevant category. If studies included control conditions, the comparators for the primary endpoint were categorized according to a coding scheme (Supplemental Material C). Proprietary names were replaced with common names (e.g. COMP360 was renamed to psilocybin). In five cases, the intervention model that was selected by the authors of the protocols in the ‘Design details’ section was corrected due to contradictions between and within the subsections ‘Participant group/arm’ and/or ‘Study overview’ in the corresponding pre-registration protocols on ClinicalTrials.Gov.

### Analysis

Descriptive analyses summarized the current landscape. To assess trends in pre-registration frequencies over time, a negative binomial regression analysis was chosen. The analysis was performed in R (version 4.4.1; R Core Team, 2024) using the glm.nb() function from the MASS package (Venables and Ripley, 2002). The significance threshold was set at *p* < 0.05.

## Results

A total of 241 pre-registration protocols met the inclusion criteria (Figure 1). The raw data are included in the Supplemental Material.

### Global trends in clinical psychedelic research

#### Intervention substances, study registration and completion over time

**Figure 2.**
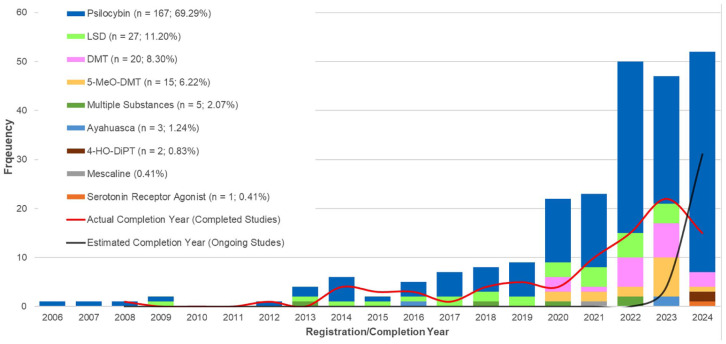
Registration and completion of clinical trials with psychedelics. For the year 2024, protocols published up to 11 November 2024 were considered, as this was the day of the search. Bars represent the number of trials registered per year, colour-coded by substance. The lines indicate the completion status: actual completion years for completed studies (up to when the search was conducted) and estimated completion years for currently ongoing studies. Both lines are delayed relative to the registration years, reflecting the time required to conduct and finalize studies. The substance investigated in the clinical trial mentioning Serotonin Receptor Agonist is the psychedelic substance GM-2505, a short-acting 5-HT2A receptor agonist/5-HT releaser. *N* = 5 trials investigated more than one serotonergic psychedelic substance and aimed to compare the acute subjective or pharmacological effects of different compounds in healthy participants; *N* = 4 were classified as (Early) phase I, and *N* = 1 was labelled ‘Not Applicable’, indicating no FDA-defined trial phase. Among all these, *n* = 4 used a within-subject crossover design (NCT04227756, NCT03604744, NCT05570708, NCT05523401), and *n* = 1 used a between-subject design (NCT02033707). Substances studied across these trials include lysergic acid diethylamide (LSD), a widely used classical long-acting psychelic, psilocybin, mescaline, N,N-dimethyltryptamine (DMT ), a short-acting psychedelic, and 4-bromo-2,5-dimethoxyphenethylamine (2C-B).

On average, *M* = 12.68 (standard deviation (SD) = 17.72) studies were registered annually from 2006 to 2024. Registrations increased by 312.8% from 2006–2019 (*n* = 47; 19.50%) to 2020–2024 (*n* = 194; 80.50%). The number of protocols increased by a factor of 2.44 from 2019 to 2020, reflecting the largest annual rise. Growth continued into 2021 and rose 2.17-fold in 2022 (Figure 2).

The negative binomial regression indicated an exponential increase in study registrations over time (β = 0.218, *p* < 0.001), with an average annual increase of 24.36%. Additionally, there was a significant rise in registrations post-2019 (β = 0.689, *p* = 0.017), indicating that beyond the general time trend, the registration rate increased by 99.17% in the post-2019 period compared to pre-2019 levels (Supplemental Material D).

According to estimated completion dates reported in protocols for planned or ongoing studies, *n* = 59 trials are scheduled to complete in 2025; *n* = 28 in 2026; *n* = 19 in 2027; *n* = 8 in 2028; and *n* = 4 in 2029. Numbers represent a lower bound, as more protocols are expected to be registered in the near future. The frequency of investigated substances is depicted in Figure 2.

#### Study status

Of all protocols, 36.51% (*n* = 88) were completed, whereas 63.49% (*n* *=* 153) were still active: 30.83% (*n* = 80) recruiting, 20.33% not yet recruiting (*n* = 49), 8.71% (*n* = 21) active not recruiting anymore, and 3.61% (*n* = 3) are enrolling by invitation (category definitions are found in Supplemental Material E). Of completed studies, the mean duration (time between study start and completion) was *M* = 30.97 months (SD = 30.18), ranging from 1.28 to 181.97 months (Mdn = 20.52 months).

#### Study phase and primary purpose

**Figure 3.**
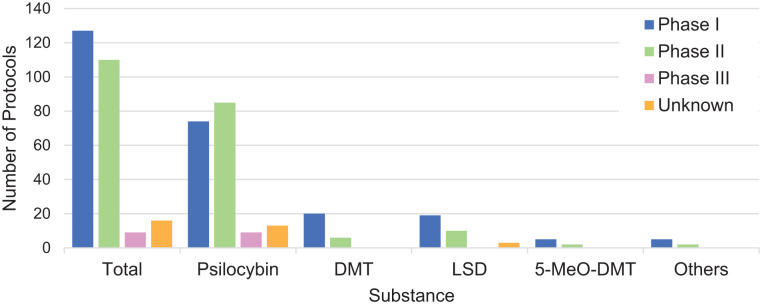
Study phases of clinical trials per psychedelic substance. Early phase I was included in the phase I category. If a combination of two phases was mentioned, the protocol was classified under the earlier phase (e.g. phase I and phase II were classified as phase I). Additional details are listed in the PRISMA chart (Figure 1). After the search was conducted, a phase III study registration investigating the effects of lysergic acid diethylamide (LSD), a widely used classical psychedelic substance, on generalized anxiety disorder was published, which is not included in this analysis.

Figure 3 shows trial phases by substance. Indications targeted in phase III trials, which only included psilocybin as intervention substance, were depressive disorders, including major depressive disorder and treatment-resistant depression (*n* = 8), and anxiety, depression and existential distress in advanced cancer (*n* = 1).

#### Sponsors

**Figure 4.**
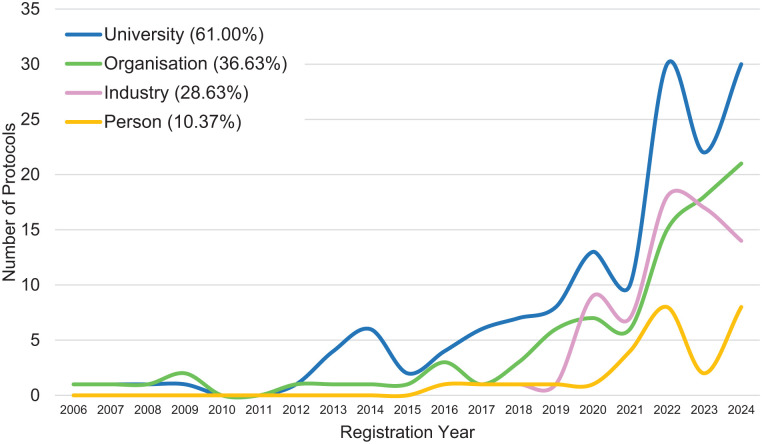
Sponsors of clinical trials involving psychedelic substances. The sum of all percentages exceeds 100%, as *n* = 87 (36.1%) of clinical trial protocols are sponsored by a collaboration of ⩾2 institutions.

Sponsor is defined as the entity that initiates and has authority over the study (U.S. National Library of Medicine, n.d.). Universities sponsored *n* = 147 studies (61%), independently or through collaborations, with University Hospital, Basel, Switzerland (*n* = 23) and Johns Hopkins University (*n* = 22) as top sponsors. Non-profit organizations were involved in *n* = 89 studies (36.93%), led by Centre for Addiction and Mental Health and NYU Langone Health (each *n* = 5). For-profit, industry-involvement was found in *n* = 69 studies (28.63%), led by GH Research Ireland Limited (*n* = 8) and COMPASS Pathways (*n* = 7). Individual persons contributed to *n* = 25 studies (10.37%; Figure 4).

Geographically, *n* = 61.83% (*n* *=* 149) of sponsors were located in North America, 35.68% in Europe (*n* *=* 86), 1.24% in South America (*n* *=* 3) and 0.41% each in Asia, Australia and New Zealand (each *n* *=* 1).

#### Study sites

**Figure 5.**
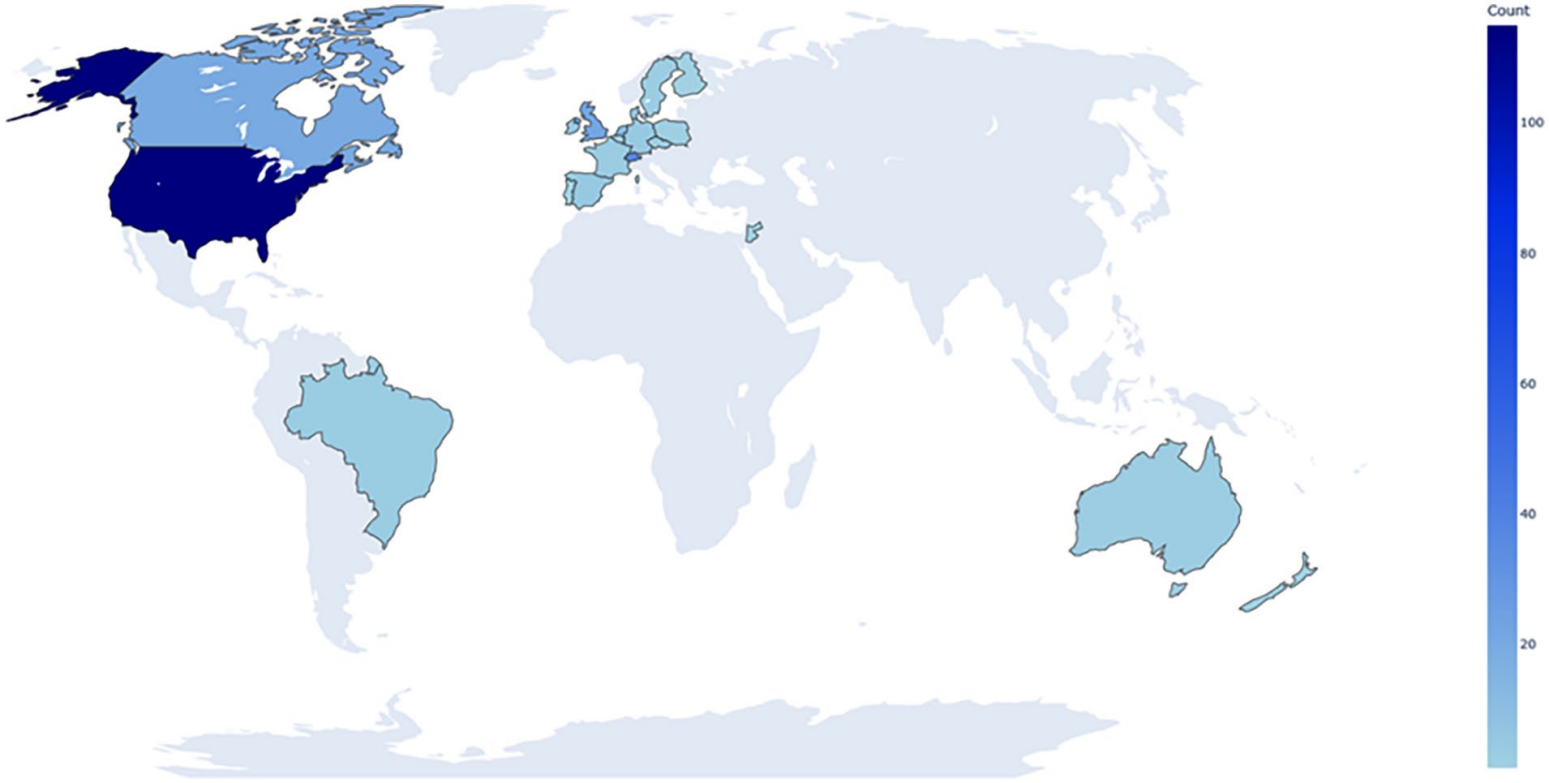
Geographic distribution of study sites for clinical trials involving psychedelic substances. The majority of studies were conducted in the United States (*n* = 115, 41.82%), followed by Switzerland (*n* = 37, 13.45%), the United Kingdom (*n* = 22, 8%), Canada (*n* = 20, 7.27%) and the Netherlands (*n* = 12, 4.36%). Other countries with notable representation include Denmark (*n* = 6, 2.18%), Germany, Ireland and Spain (each *n* = 5, 1.82%), and Brazil and France (each *n* = 4, 1.45%). Fewer studies were conducted in Australia, Czechia and Sweden (each *n* = 3, 1.09%), and Belgium and Poland (each *n* = 2, 0.73%). Finland, Jordan, New Zealand and Portugal each accounted for one study (0.36%). Additionally, 23 studies (8.36%) did not report location data. The colour intensity corresponds to the count of studies per country, with darker shades indicating higher counts.

Studies were conducted across 20 countries (Figure 5). Overall, 16.60% (*n* *=* 40) of studies involved multiple locations, with a mean of 9.38 locations per study (SD = 15.33, Mdn = 2.5, range = 2–77). Among those, 12.03% (*n* *=* 29) were conducted at multiple sites within 1 country, and 11 trials were multi-national. In 9.54% (*n* *=* 23) of research protocols, no information about study location was specified.

### Study characteristics

#### Study populations

The mean (estimated) sample size was *M* = 49.99 (SD = 59.4), with a median of Mdn = 30 (range: 5–568). Most protocols allowed all sexes (*n* = 231, 95.85%), while a smaller subset was male-only (*n* = 4, 1.66%) or female-only (*n* = 6, 2.49%). The minimum age for participation ranged from 16 to 60 years (Mdn = 19), and the maximum age ranged from 25 to 100 years (Mdn = 65).

**Figure 6.**
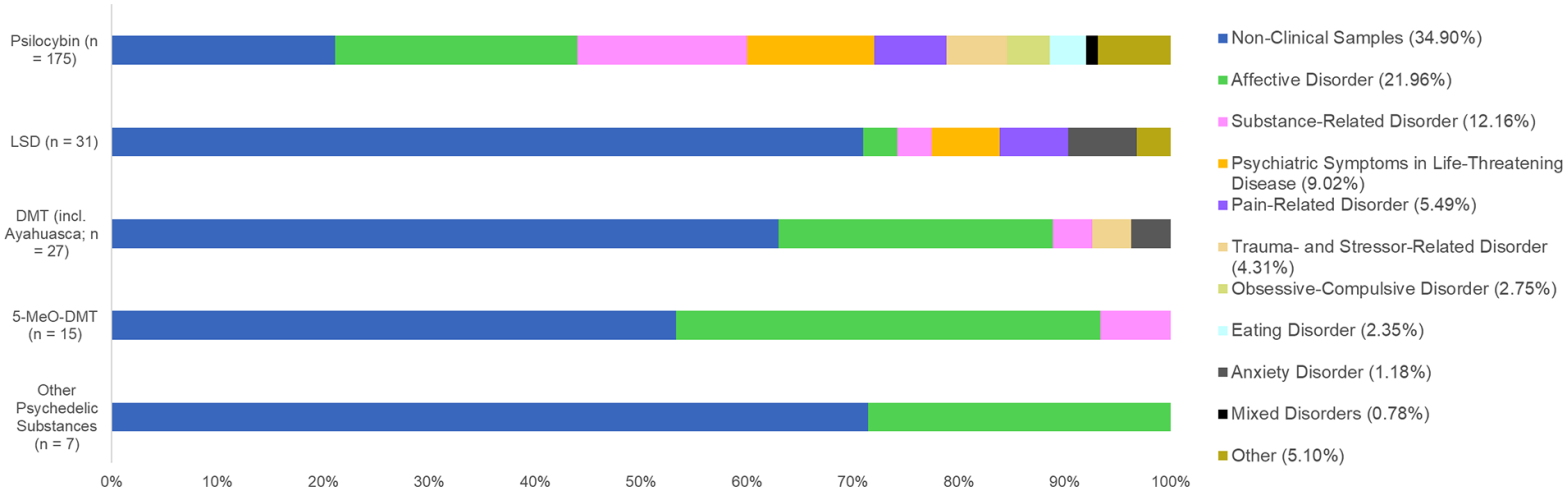
Study samples and conditions targeted within clinical populations per psychedelic substance. Within the clinical samples, the conditions targeted were: affective disorders (*n* = 56; including treatment-resistant, postpartum, major depression and bipolar II), substance-related disorders (*n* = 31; including alcohol, nicotine, cocaine, methamphetamine and opioid use disorders, with 2 studies targeting comorbid depressive symptoms), psychiatric symptoms in life-threatening diseases (*n* = 23; including pain, demoralization, depressive and anxiety symptoms in (advanced) cancer, amyotrophic lateral sclerosis (ALS), a progressive neurodegenerative disease that destroys motor neurons and causes muscle paralysis, or Acquired Immunodeficiency Syndrome (AIDS), the advanced stage of human immunodeficiency virus infection, marked by severe immune system failiure), pain-related disorders (*n* = 14; including cluster headache, migraine, fibromyalgia, phantom limb pain (lower), back pain and neuropathic pain, with 2 studies targeting comorbid depressive symptoms and 1 targeting co-morbid opioid dependence), trauma- and stressor-related disorders (*n* = 11; including post-traumatic stress disorder (PTSD), a psychiatric disorder caused by a traumatic experience, and grief, with 3 studies targeting comorbid depressive symptoms), obsessive-compulsive disorder (*n* = 7), eating disorders (*n* = 6; including anorexia nervosa and binge eating disorder), anxiety-related disorders (*n* = 3; i.e. including generalized anxiety disorder), mixed disorders (*n* = 2; inclusion of multiple psychiatric disorders) and other conditions (*n* = 13; including attention-deficit hyperactivity disorder (ADHD), a neurodevelopmental disorder characterized by inattention, impulsivity, and hyperactivity, autism spectrum disorder, body dysmorphic disorder, Lyme disease, persistent post-concussion symptoms, psychogenic nonepileptic seizures, irritable bowel syndrome, post-treatment Lyme disease, functional neurological disorder, borderline personality disorder and mild cognitive impairment or early Alzheimer’s disease (three studies targeting comorbid depressive and/or anxiety symptoms)).

Non-clinical subjects (i.e. healthy participants) were targeted in 81 cases (32.79%), while clinical populations were targeted in 166 protocols (67.21%; Figure 6). The total exceeds the number of included protocols due to three protocols employing a two-study design, investigating distinct populations within sub-studies.

#### Number of active drug sessions

Most trials included a single active session (51.04%, *n* = 123), while 33.20% (*n* = 80) implemented multiple fixed sessions (with most specifying 2 sessions (*n* = 53), and fewer specifying 3 (*n* = 7), 4 (*n* = 9), 5 (*n* = 4), or more sessions (*n* = 7)). Flexible session counts were used in 10.79% (*n* = 26) of trials, permitting additional sessions (e.g. open-label extensions or if remission was not achieved), with most permitting one extra session (*n* = 21, 8.71% of all studies). In 4.98% (*n* = 12) of trials, the number of active sessions was unspecified. Dosing and session details are shown in Table 1.

**Table 1.** Study characteristics per psychedelic substance.

Psychedelic Substance	Psilocybin (*n* = 172)		LSD (*n* = 31)		DMT^a^ (*n* = 24)		5-MeO-DMT (*n* = 15)		Mescaline (*n* = 3)		Others^b^ (*n* = 4)	
	*M* (SD)	Mdn (Min-Max)	*M* (SD)	Mdn (Min-Max)	*M* (SD)	Mdn (Min-Max)	*M* (SD)	Mdn (Min-Max)	*M* (SD)	Mdn (Min-Max)	*M* (SD)	Mdn (Min-Max)
Fixed dosing regimen, *N* (%)	123	71.51	30	96.77	7	29.17	6	40.00	2	66.67	3	75.00
Minimum dose	20.04 mg (8.53)	25 mg (0.25–30)	84.32 μg (57.41)	100 μg (5–200)	33.16 mg (24.3)	39.3 mg (5–60)	5.08 mg (2.24)	6 mg (0.5–6)	200 mg (141.42)	200 mg (100–300)	23.33 mg (11.55)	30 mg (10–30)
Maximum dose	23.73 mg (7.57)	25 mg (2–50)	121.23 μg (70.04)	100 μg (20–250)	82.19 mg (65.28)	60 mg (25–216)	17.17 mg (2.04)	18 mg (13–18)	650 mg (212.13)	650 mg (500–800)	30 mg (0)	30 mg (30–30)
Relative dosing regimen^c^	22	12.79%			5	20.83%						
Minimum dose	16.55 mg (8.06)	18.75 mg (0.15–30)			23.38 mg (45.84)	25 mg (0.1–105)						
Maximum dose	23.11 mg (9.46)	25 mg (1.5–42)			30.12 mg (46.57)	14.83 mg (0.75–112)						
Unknown	27	15.70%	1	3.23%	12	50.00%	9	60.00%	1	33.33%	1	25.00%
	*N*	%	*N*	%	*N*	%	*N*	%	*N*	%	*N*	%
Route of administration												
Oral	110	63.95	22	70.97	5	20.83			3	100		
Intravenous	2	1.16			10	41.67	1	6.67				
Intramuscular							1	6.67			2	50
Inhalation					3	12.5	7	46.67			1	25
Nasal							5	33.33				
Mixed			1	3.23	1	4.17						
Not specified	60	34.88	8	25.81	5	20.83	1	6.67			1	25
Number of sessions												
Fixed number	142	82.56	27	87.10	19	79.17	13	86.67	2	66.67	4	100.00
One	90	52.33	10	32.26	12	50.00	12	80.00	1	33.33	2	50.00
Two	40	23.26	10	32.26	4	16.67					1	25.00
Three	4	2.33	1	3.23			1	6.67			1	25.00
Four	4	2.33	2	6.45	1	4.17						
More than four	4	2.33	4	12.90	2	8.33			1	33.33		
Multiple flexible number (i.e. optional extension)	21	12.21	2	6.45	5	20.83			1	33.33	0	0.00
Unknown	9	5.23	2	6.45			2	13.33				
Comparator conditions												
No comparator	63	36.63	2	6.45	1	4.17	4	26.67			1	25.00
Non-psychedelic, non-psychoactive substance	36	20.93	22	71.00	11	45.83	7	46.67	2	66.67	1	25.00
Psychedelic: different dose	19	11.05	5	16.13	7	29.17	4	26.67			2	50.00
Non-psychedelic, psychoactive substance	28	16.28	1	3.23	1	4.17			1	33.33		
Psychedelic + augmented intervention	9	5.23	1	3.23	1	4.17						
Waitlist	7	4.07										
Psychological/behavioural comparator	5	2.91			1	4.17						
Psychedelic: Different frequency, sequence, or route of administration	2	1.16			1	4.17						
Other	3	1.74			1	4.17						
Intervention model												
Parallel groups	77	44.77	10	32.26	11	45.83	5	33.33	1	33.33	2	50.00
Single group	67	38.95	4	12.90	1	4.17	4	26.67			1	25.00
Crossover	18	10.46	17	54.84	10	41.67	1	6.67	2	66.67	1	25.00
Sequential	7	4.07			2	8.33	5	33.33				
Factorial	3	1.74										

The sum of all substances exceeds the total number of protocols, as protocols investigating multiple substances were counted multiple times (for each individual substance).

LSD: lysergic acid diethylamide, a serotonergic long-acting psychedelic; DMT: N,N-dimethyltryptamine, a short-acting psychedelic contained e.g., in Ayahuasca; 5-MeO-DMT: 5-methoxy-N,N-dimethyltryptamine, a short-acting psychedelic; TAU: treatment as usual; SD: standard deviation.

a Including Ayahuasca (*n* = 3).

b Including 2C-B (*n* = 1), 4-HO-DiPT (*n* = 2), and a not further specified psychedelic serotonin receptor agonist (*n* = 1).

c Relative to body weight in kilogram. All numbers were multiplied by 70, so indicated numbers refer to a average person with a body weight of 70 kg. Non-psychedelic, non-psychoactive substances included, for example, mannitol or lactose. Psychedelic Comparators were subdivided into categories: different doses of the same psychedelic; variations in frequency, sequence, or administration route. Non-psychedelic, psychoactive substances included, for example, niacin, methylphenidate, tetrahydrocannabinol (THC), the main psychactive substance found in cannabis. Psychedelics with augmented interventions included, for example, Psychedelic + Ketanserin/Escitalopram/Mindfulness Meditation versus psychedelic only. Psychological/Behavioural Comparators comprised interventions like breathwork or TAU only. Some trials utilized Waitlist controls. The Other category includes multiple comparators combining different comparators (factorial study designs). More information can be found in Supplemental Material C.

#### Comparator conditions

Of all retrieved protocols, 70.54% (*n* *=* 170) included a comparator condition, while 29.46% (*n* *=* 71) did not include any comparator (detailed information in Table 1 and Supplemental Table C1).

#### Previous experience with psychedelic substances

Previous psychedelic experience was allowed in 56.02% (*n* *=* 135) of protocols, required in 6.22% (*n* *=* 15), and not allowed (i.e. participants naïve to psychedelics) in 3.73% (*n* *=* 9) of protocols. Four studies (1.66%) had mixed requirements for different sub-populations within one study, while 32.37% (*n* *=* 78) provided no information on nativity requirements.

#### Continuation of serotonin reuptake inhibitors

Serotonin reuptake inhibitors (SERT; formerly SSRIs) use during participation was prohibited in 63.49% (*n* = 153), permitted in 6.22% (*n* = 15) and required in 0.83% (*n* = 2) of cases. Four studies (1.66%) had mixed requirements for sub-populations of the same study. Additionally, 27.8% (*n* = 67) did not provide information on SERT use.

### Psychotherapy component

A psychotherapy component was explicitly reported in 52.7% (*n* = 127), however, most protocols (*n* = 68) mentioned generic terms like ‘supportive conditions’ or ‘(substance-assisted) psychotherapy’. Among explicitly named approaches, most were non-standardized interventions and included: cognitive behavioural interventions (*n* = 12), group therapy (*n* = 7), *Set-and-Setting* Approach (*n* = 7), motivational enhancement therapy (*n* = 5), psychoeducation (*n* = 4), mindfulness therapy (*n* = 3), palliative care support (*n* = 3), acceptance and commitment therapy (or versions thereof; *n* = 3) and one instance of constructivist therapy, interpersonal therapy, trauma-focused therapy and psilocybin-assisted existential, attachment and relational therapy. Among protocols reporting psychotherapy, 57.48% (*n* = 73) included preparatory sessions, 51.97% (*n* = 66) integration sessions, and 48.82% (*n* = 62) explicitly mentioned both. Only 33 protocols provided details on session numbers: mean number of preparation sessions/hours was *M* = 3.24 sessions/hours (SD = 1.58, range: 1–8), whereas the mean number of integration sessions/hours was *M* = 3.88 (SD = 2.47, range: 1–12). Most protocols did not clarify whether values referred to hours or sessions.

To assess changes in psychotherapy reporting, all 79 pre-registered clinical trial protocols published after the FDA’s June 2023 recommendation were compared with 79 pre-registration protocols published before the recommendation (U.S. Food and Drug Administration, 2023): A numerical increase in the reporting of psychotherapy components from 51.90% (*n* = 38) to 60.76% (*n* = 48) was observed. Despite a numerical decline in the average number of preparation and integration sessions/hours (7.09 vs 5.29 sessions/hours), the proportion of the protocols reporting specific numbers numerically increased from 13.92% (*n* = 11) to 21.52% (*n* = 17). No qualitative changes were observed regarding the reporting of applied therapeutic approaches.

## Discussion

This landscape analysis summarized 241 clinical trial pre-registrations involving classical, serotonergic psychedelics (i.e. 5-HT2A agonists) on ClinicalTrials.Gov published from 2006 to November 2024, demonstrating exponential growth over the past two decades, particularly after 2019, with 80.5% of registrations occurring between 2020 and 2024. The post-2019 surge coincides with and is likely fuelled by promising early results, regulatory advancements (e.g. FDA ‘breakthrough therapy’ designations), and institutional milestones (establishment of the Centre for Psychedelic Research at Imperial College London; Center for Psychedelic and Consciousness Research at Johns Hopkins University (Aday et al., 2020; Johns Hopkins Medicine, 2019; O’Hare, 2019). Psilocybin is most advanced towards clinical approval and remains dominant in research trials, accounting for over two-thirds of protocols and targeting the widest range of conditions. However, the field is rapidly evolving, and other substances are also progressing. Shortly after the current search was conducted, the first phase III trial with LSD for generalized anxiety disorder was pre-registered on ClinicalTrials.Gov.

Two-thirds of trials are ongoing, with peak completions expected in 2025. Approximately two-thirds investigate therapeutic effects in over 10 clinical populations, largely focusing on affective, substance-related disorders, and psychiatric symptoms in life-threatening diseases. Research is concentrated in Western, industrialized, educated, democratic, high-income regions (i.e. North America and Europe). Although pre-registration databases do not provide standardized information on participants’ cultural, socioeconomic or sexual identity, it is likely that most trial populations are composed of white, cisgender individuals from mid- to high-income backgrounds. This assumption is supported by research showing that women, queer individuals, Indigenous people, people of colour and individuals from the Global South are underrepresented in psychedelic research participation, compromising generalizability across diverse populations (Hovmand et al., 2023; Labate et al., 2025).

The psychedelic research landscape is marked by substantial heterogeneity, not only encompassing numerous substances, indications and trial designs, with only a few indications under investigation in advanced-phase trials, but also in dosing regimens, administration schedules and comparator conditions. The heterogeneity might reflect the historical trajectory of the field, which has largely been shaped by university-led research. Unlike industry-driven studies, which often focus on a single substance and indication with the clear goal of regulatory approval, previous academic research has been characterized by a more heterogeneous approach, investigating the potential therapeutic effects of psychedelics across a broad range of mental health conditions simultaneously (Norring and Spigarelli, 2024).

Differences in doses, number of sessions or use of active versus inactive controls, in- or exclusion of research subjects with prior experience with psychedelics and concomitant SERT use likely influence both effect sizes and safety profiles (Hovmand et al., 2023; Muthukumaraswamy et al., 2021). While some degree of variation is expected across trial phases, our analysis revealed substantial heterogeneity even among trials targeting similar indications with the same substance. Lack of standardization in comparator conditions (e.g. active vs inactive placebo, waitlist control) can compromise blinding integrity, enhance expectancy effects and introduce interpretive ambiguity, potentially leading to over- or underestimation of therapeutic effects of psychedelic substances. Without harmonization, or at least transparent reporting, of these methodological parameters and their potential implications, cross-trial comparisons and meta-analytic synthesis will remain limited.

Since 2019, the increasing involvement of industry has further shaped the field, accelerating clinical development by mobilizing resources, focusing efforts on a smaller number of substances and indications, and aligning studies more closely with regulatory pathways. However, industry involvement might direct research towards indications with strong market potential, potentially neglecting less profitable but socially relevant conditions. Industry-sponsored studies have been shown to more frequently report efficacy outcomes and conclusions aligned with company interests compared to non-industry-sponsored trials. This may result from trial design choices, such as the selection of comparators or dosing frequency, as well as selective and/or non-transparent outcome reporting (Lundh et al., 2017). These risks are especially relevant in psychedelic research, where expectations and subjective outcomes are highly susceptible to bias. Proprietary treatment protocols (e.g. ‘Set-and-Setting Approach’) and restricted data access may further limit transparency and hinder independent validation. To safeguard scientific integrity and maintain public trust, clear reporting standards starting at pre-registration stages, independent replication and continued public-interest research funding remain essential (Holman and Elliott, 2018; Lundh et al., 2017).

While early research primarily focused on long-acting psychedelics (LSD, psilocybin), interest in shorter-acting compounds has increased since 2020. For example, 5-MeO-DMT’s psychoactive effects last 45–90 minutes (Rucker et al., 2024); compared to psilocybin’s 5.6–6.4 hours (Holze et al., 2023). This approximately fivefold reduction might offer a more resource-efficient option for clinical practice. First results show promising effectivity with mild, self-resolving adverse events (Reckweg et al., 2023). More clinical trials are required to test its effectiveness and safety compared to longer-acting psychedelics. This shift towards shorter-acting substances may reflect the growing influence of industry sponsors and economic considerations. Shorter session durations could reduce costs and facilitate integration into mainstream mental health care models, but also raise questions about how much therapeutic depth, and thus, long-term treatment efficacy, is preserved.

The role of psychotherapy in psychedelic research, particularly its necessity and impact on therapeutic effects, is a major topic of debate (Goodwin et al., 2024). Half of protocols report the use of psychotherapeutic interventions, often mentioning broad, unspecific (e.g. ‘supportive conditions’) or non-standardized approaches (e.g. ‘Set-and-Setting Approach’). Following the FDA’s 2023 recommendations (U.S. Food and Drug Administration, 2023), reporting of psychotherapy numerically increased in quantity, but the quality of descriptions of applied interventions remained largely vague and unchanged. The observed numerical decline in preparation and integration sessions suggests a trend towards streamlining, potentially reflecting raising industry involvement and its feasibility and cost-efficiency interests.

Insufficient reporting of psychotherapeutic components reduces comparability across studies and complicates efforts to identify which combinations of psychedelic compounds and therapeutic approaches (e.g. intervention, intensity, timing) yield the most effective outcomes. This challenge could be addressed through standardized reporting frameworks implemented at the pre-registration stage (Brennan et al., 2023). Unlike typical pharmacological treatments, therapy with psychedelics typically integrates non-pharmacological components. The absence of a dedicated section for psychotherapy components in ClinicalTrials.Gov, relying instead on free-text fields, complicates standardized reporting during trial registration. The implementation of structured templates for non-pharmacological components, such as the Template for Intervention Description and Replication (TIDieR) checklist (Hoffmann et al., 2014), could enhance reproducibility and comparability. The TIDieR provides 12 essential items for describing non-pharmacological interventions, including: the name and rationale of the intervention, materials used, procedures, provider characteristics and training, mode of delivery (e.g. group vs individual), setting, duration and intensity, tailoring, any modifications, and strategies to assess and maintain fidelity, along with reporting of actual adherence. These elements are highly relevant for psychedelic-assisted therapies, which typically involve structured psychotherapeutic components across preparation, administration and integration. Integrating these elements at the pre-registration stage could meaningfully improve methodological transparency, facilitate future evidence synthesis and reduce interpretive bias in this emerging field (Muthukumaraswamy et al., 2021; Van Elk and Fried, 2023). As the field continues to evolve, methodological harmonization, equitable research practices, and rigorous transparency will be key to translating early promise into safe, effective and accessible psychedelic therapies.

### Limitations

This analysis focused exclusively on ClinicalTrials.Gov, the largest pre-registration database, which may have overrepresented Western trials due to U.S. registration obligations. Given the predominance of high-income, Western trials and lack of participant-level diversity data, our findings reflect the research activity and design choices typical of these contexts. Our focus on ClinicalTrials.Gov was motivated by its broad international coverage, structured data fields, and consistent use in regulatory and academic contexts. However, we acknowledge that this approach excludes studies registered only in regional databases. A search on the WHO’s International Clinical Trials Registry Platform (World Health Organization, 2018) was performed for cross-validation and resulted in a total of *N* = 154 records (compared to *N* = 975 searching only ClinicalTrials.Gov). One additional clinical trial was retrieved that was registered in Japan (Uchida, 2023) and is not included in the current analysis. The Australian New Zealand Clinical Trials Registry (ANZCTR – Registration, n.d.) resulted in additional results that are not included in this analysis. As these studies are not cross-registered on ClinicalTrials.Gov, their exclusion may result in underrepresentation of research activity in Australia and New Zealand; regions with increasing relevance following recent regulatory and funding developments. This limits the geographic generalizability of our findings.

Data quality depends on the completeness and accuracy of reviewed protocols. Issues with pre-registration data involve a large heterogeneity in details of information in free-text input fields, within-protocol inconsistencies due to a lack of input constraints, and insufficient updating of the protocols (Hartung et al., 2014; Talebi et al., 2020). Similarly, not all clinical trials involving psychedelic substances are pre-registered (Hovmand et al., 2023), possibly compromising the completeness of this landscape analysis.

As many new protocols avoid the term ‘psychedelics’, search results depend on the completeness of search terms. Extensive efforts were made to identify all relevant proprietary names and ensure comprehensive data retrieval; however, it cannot be ruled out that not all brand names were captured.

## Conclusion

This analysis highlights the exponential rise in psychedelic clinical trial registrations since 2006, with a substantial acceleration starting in 2019. Most studies are still ongoing, signalling a forthcoming surge in findings. While research continues to explore diverse substances, clinical indications and administration methods, recent trends show a shift towards shorter-acting psychedelics and a narrower clinical focus, coinciding with increased industry involvement. Despite ongoing discourse in the field, the representation of psychotherapeutic components in pre-registration protocols remains limited. Although newer protocols more frequently acknowledge non-pharmacological interventions, their descriptions often lack detail and specificity. Future efforts should focus on harmonizing the reporting of psychotherapy elements already at trial planning stages, by adapting structured frameworks such as the TIDieR checklist (Hoffmann et al., 2014), which provide guidance for the standardized description of complex interventions. Adding data input constraints as well as fields for non-pharmacological treatment elements to CilinicalTrials.Gov could enhance future completeness and consistency within protocols. To advance the field responsibly, future pre-registration practices should include structured, standardized reporting of both pharmacological and psychotherapeutic components, along with key design choices such as comparator selection, blinding strategies and inclusion criteria. Clearer documentation at trial planning stages can improve methodological transparency, reduce interpretive ambiguity, and facilitate more meaningful cross-trial comparisons.

## Supplemental Material

sj-docx-1-jop-10.1177_02698811251371690 – Supplemental material for Landscape analysis of pre-registered clinical trials involving classical psychedelics Supplemental material, sj-docx-1-jop-10.1177_02698811251371690 for Landscape analysis of pre-registered clinical trials involving classical psychedelics by Abdo Uyar, Linda Forbrich, Ulrike Lueken and Ricarda Evens in Journal of Psychopharmacology
