# Supplementary material for: Landscape analysis of pre-registered clinical trials involving classical psychedelics
Source: J Psychopharmacol. 2025 Oct 21;40(5):720–31. doi: 10.1177/02698811251371690 (PMC13310267; doi:10.1177/02698811251371690)
Supplement: sj-docx-1-jop-10.1177_02698811251371690 – Supplemental material for Landscape analysis of pre-registered clinical trials involving classical psychedelics [file sj-docx-1-jop-10.1177_02698811251371690.docx]

# **Landscape Analysis of Pre-Registered Clinical Trials involving Classical Psychedelics: Supplementary Material**

**Table of Contents**

[**Supplementary Material A** 1](#_Toc191562133)

[**Supplementary Material B** 2](#_Toc191562134)

[**Supplementary Material C** 3](#_Toc191562135)

[**Supplementary Material D** 5](#_Toc191562136)

[**Supplementary Material E** 8](#_Toc191562137)

# **Supplementary Material A**

**Search Term**

psychedelic* OR hallucinogen* OR Psilocybin OR LSD OR Lysergic Acid Diethylamide OR DMT OR Dimethyltryptamine OR n,n-Dimethyltryptamine OR Ayahuasca OR 5-MeO-DMT OR 5 Methoxy N,N-Dimethyltryptamine OR Mescaline OR 2C-B OR 4-Bromo-2,5-Dimethoxyphenethylamine OR COMP360 OR PSIL201 OR PSIL102 OR MEO101 OR GH001 OR GH002 OR GH003 OR MM-120 OR SYNP-101 OR APEX-52 OR APEX-90 OR NM-1001 OR BPL-003 OR BPL-002 OR ELE-101 OR ELE-Psilo+ OR ELE-Psilo OR LPH-5 OR LPH-48 OR TRP-8802 OR TRP-8803 OR PSX-001 OR PEX010 OR CYB003 OR CYB004 OR CYB001 OR SPL028 OR SPL026 OR BMND01 OR BMND02 OR BMND03 OR BMND05 OR BMND06 OR BMND08 OR BMND09 OR MPS-1014 OR MSP-4019 OR MSP-4020 OR MSP-1014 OR L-130 OR JOUR-5700 OR GM-2505 OR RSTP-1000 OR VLS-01 OR RE01 OR RE02 OR Psylo-3001 OR Psylo-4001 OR Psylo-5001 OR PLZ-1015 OR O-acetylpsilocin OR 4-AcO-DMT OR LSR-1019 OR Lucid-201 OR EB-002 OR BMB-202 OR BMB-101 OR AKO-002 OR AKO-004 OR CMND-100 OR BOL-148 OR NYPRG-101 OR BETR-001 OR TD-0148A OR EBRX-101 OR PSY-0.1 OR PSY-0.2 OR PSY-0.3 OR PSY-0.4 OR PSY-0.5 OR PSY-0.6 OR RE-104 OR FT-104 OR 4-HO-DiPT OR iprocin prodrug OR RE104 OR TRP-8802 OR TRP-8803 OR TRP-8804 OR TRYP-0082 OR PEX-100 OR MYCO-001 OR MYCO-003 OR MB-22001.

# **Supplementary Material B**

Data downloaded directly from ClinicalTrials.gov included: study URL, title, identifier, status, sponsor type, name, region, and country, collaborators, study location(s), submission date, last update, brief and detailed description, (estimated) start date, (estimated) primary completion date, (estimated) completion date, (estimated) sample size, study phase, primary purpose, conditions/indications, minimum and maximum eligible ages, sexes, intervention substance, allocation, intervention model, and outcome measure(s).

Variables extracted utilizing a two-extractor approach included: number of substance sessions, minimum and maximum dosages, comparator conditions, psychotherapy component, and if available, number of preparation and integration sessions (if a range was provided, the higher number was extracted), and data on exclusion criteria, regarding the current use of SERT medication and lifetime psychedelic use.

# **Supplementary Material C**

**Categorisation System for Comparators**

**Table C1**

*Categorization and Explanation for Comparators*

| Category | Category Label | Explanation |
| --- | --- | --- |
| 0 | No Comparator | *NA* |
| 1 | Non-Psychedelic Psychoactive Comparator | Psychoactive substances that are not classical psychedelics (e.g. methylphenidate, THC) |
| 2 | Psychedelic Substance (Different Dose Psychedelic) | A different dose of the same psychedelic substance used in the experimental condition. |
| 3 | Psychedelic Substance (Different frequency, sequence, or route of use) | Variations including frequency, sequence, or route of administration of the same psychedelic. |
| 4 | Psychedelic Substance + Augmentation (vs. without augmented Intervention) | The same psychedelic substance as in the experimental condition but without the addition of an augmenting intervention (e.g., psychedelic + placebo instead of psychedelic + Substance/Intervention B). |
| 5 | Non-Psychedelic, Non-Psychoactive Comparator (e.g., Mannitol, Microcrystalline Cellulose, Lactose, Maltodextrin) | Non-psychoactive substances used as controls. |
| 6 | Psychological/Behavioural Comparator (Breathwork, Family Observation, TAU, Cognitive tasks) | Psychological or behavioural interventions. |
| 7 | Waitlist |  |
| 8 | Other | Multiple comparators (factorial design) |

# **Supplementary Material D**

**Exploratory Analysis: Research Protocols over the Years**

The use of a negative binominal regression was justified by the significant overdispersion observed in the raw data ^40^, with a variance-to-mean ratio of 24.76. The model included the registration year as a continuous predictor (centred around the minimum year 2006) and a binary indicator for the post-2019 period. Centring the year variable ensured that the intercept represented the baseline level of registrations.

Model diagnostics included residual plots (figure 1) and dispersion ratio checks, confirming the model's appropriateness. The fitted model was used to generate predicted curves for three scenarios: (1) pre-2019, (2) post-2019, and (3) overall time trend without the post-2019 variable (figure 2).

Exploratory analyses were conducted to test various breakpoints by calculating the Akaike Information Criterion (AIC) for each potential year. Among all years, 2019 yielded the lowest AIC score (AIC = 88.70) and was thus selected as the most appropriate breakpoint (figure 3). A likelihood ratio test showed that the model including the breakpoint (Log-Likelihood = −40.35, AIC = 88.70) significantly outperformed a simpler model that only included time (Log-Likelihood = −43.42, AIC = 94.32; χ²(1) = 6.14, p = .013). These findings indicate that the inclusion of the breakpoint significantly improved the model's ability to explain the data, highlighting a clear structural change in the trend of study registrations starting in 2019.

| **Figure 1**  *Residuals vs. Fitted Values for Negative Binomial Regression Model*  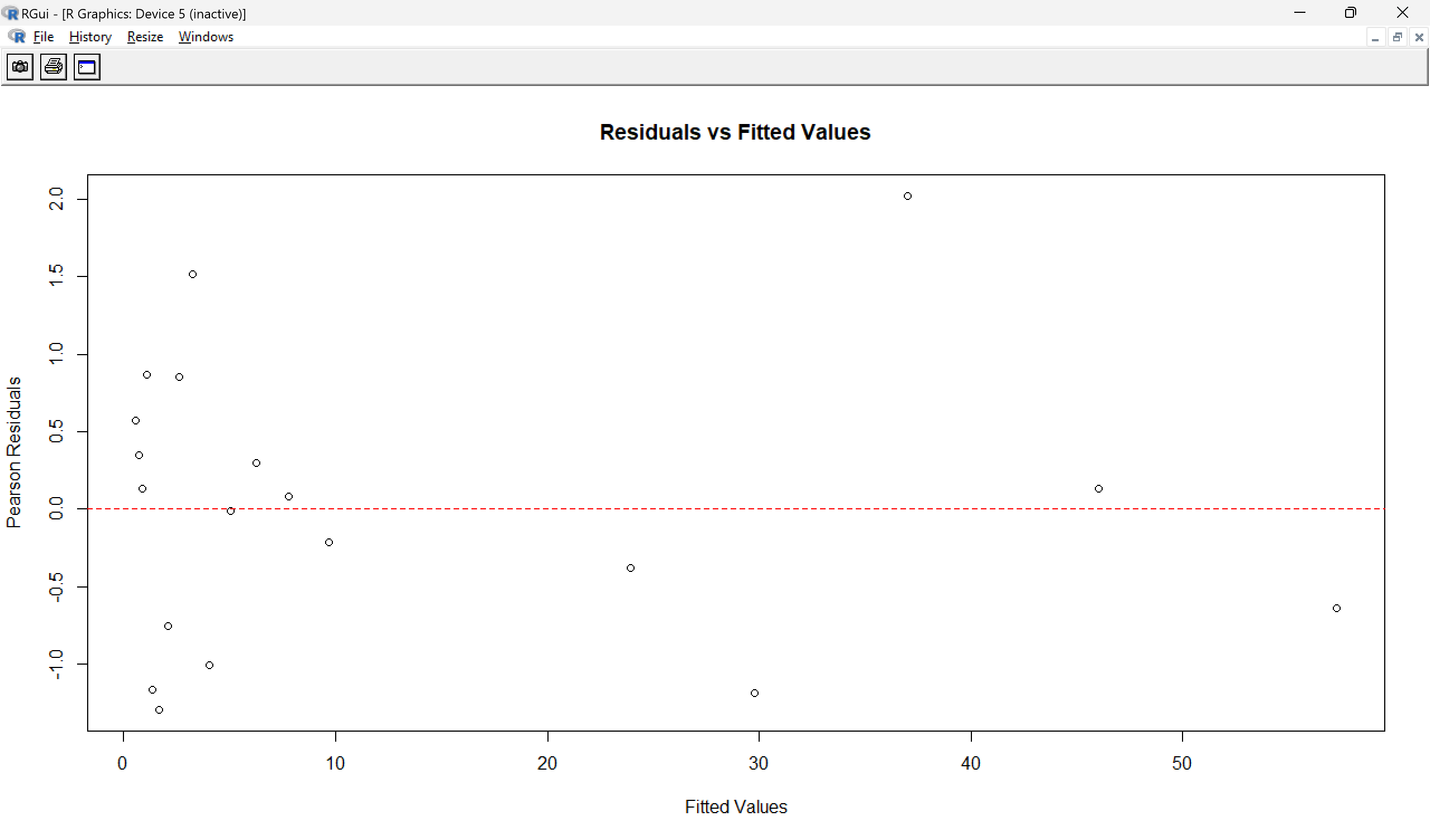  **Note**. This plot displays the Pearson residuals against the fitted values from the negative binomial regression model. The lack of a systematic pattern indicates a good model fit. The red dashed line represents the zero-residual threshold, which serves as a reference for interpreting residual deviations. |
| --- |
| **Figure 2**  *Predicted Trends in Study Registrations*   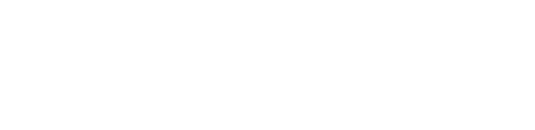 |
| ***Note***. Year Centered starts at 2006. This plot presents the predicted number of study registrations under three scenarios: pre-2019 trends (blue solid line), post-2019 trends (red dashed line), and the overall time trend without a breakpoint (green dotted line). The black dots represent observed data points, highlighting the model's fit and the distinct increase in registrations post-2019. |


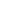

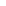

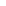

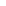

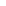

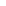

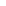


| **Figure 3**  Akaike Information Criterion (AIC) by Break Year  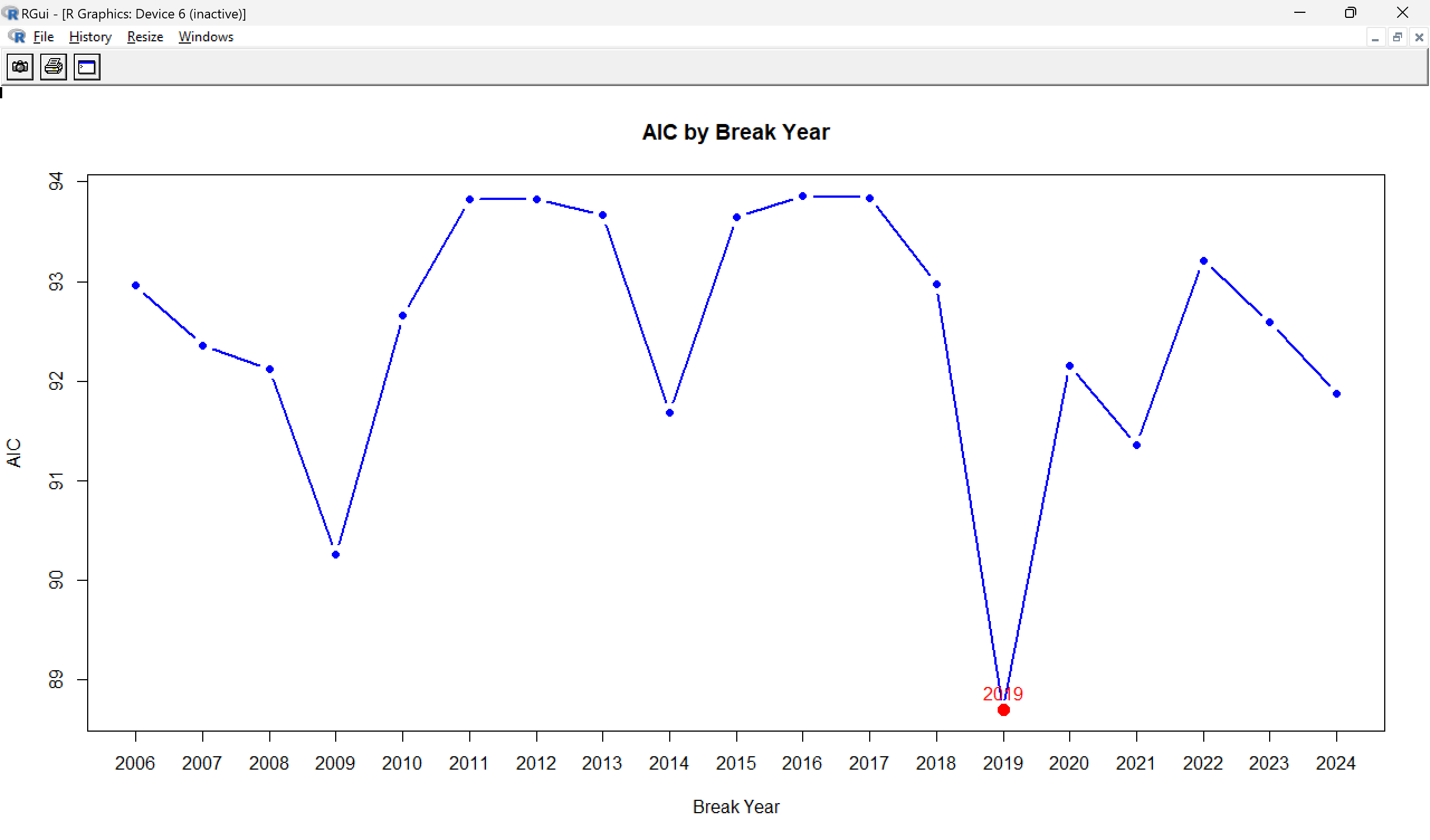 |
| --- |
| ***Note***. This plot shows the Akaike Information Criterion (AIC) values for negative binomial models with various break years. Lower AIC values indicate better model fit. The year 2019, highlighted in red, yields the lowest AIC, suggesting it as the optimal breakpoint for modelling changes in registration trends. |
|  |

# **Supplementary Material E**

**Definitions of Study Statuses**

Recruitment status categories are defined as follows: Completed (36.52%) represents concluded studies. Recruiting (30.83%) indicates studies that are actively seeking and enrolling new participants. Not Yet Recruiting (20.33%) refers to studies that have not initiated their participant recruitment phase. Active, not recruiting (8.71%) signifies ongoing studies where participants are receiving interventions or undergoing evaluations, but no new participants are being accepted. Enrolling by invitation (3.61%) refers to studies where participation is limited to pre-selected populations specifically invited by the researchers, rather than being open to all individuals^22^.
